# Acquired vulnerability against EGF receptor inhibition in gastric cancer promoted by class I histone deacetylase inhibitor entinostat

**DOI:** 10.1016/j.neo.2024.101121

**Published:** 2025-01-25

**Authors:** Tamara Zenz, Robert Jenke, Denys Oliinyk, Sandra Noske, René Thieme, Tim Kahl, Ines Gockel, Florian Meier-Rosar, Achim Aigner, Thomas RH Büch

**Affiliations:** a Leipzig University, Medical Faculty, Rudolf-Boehm-Institute for Pharmacology and Toxicology, Clinical Pharmacology, Leipzig, Germany; b University Cancer Center Leipzig (UCCL), University Hospital Leipzig, Leipzig, Germany; c Comprehensive Cancer Center Central Germany (CCCG), Leipzig and Jena; d Jena University Hospital, Functional Proteomics, Research Center Lobeda, Jena, Germany; e Department of Visceral, Transplant, Thoracic and Vascular Surgery, University Hospital of Leipzig, Leipzig, Germany; f Division of Oncology/Hematology, Cantonal Hospital Graubünden, Chur, Switzerland

**Keywords:** Gastric cancer, Entinostat, EGFR, Amphiregulin, Histone deacetylases, HDAC inhibition

## Abstract

**Introduction:**

Histone deacetylase inhibitors (HDACi) have shown promising preclinical activity in gastric cancer cells; unfortunately, however, these could not be confirmed in clinical trials. This highlights the need for the identification of underlying reasons, which may also provide the basis for possible combination therapies. Here, we delineated the effects of HDACi on components of EGFR signalling in gastric cancer cells.

**Methods:**

We investigated entinostat effects on EGFR and amphiregulin (AREG) expression in various cell line- and primary patient tumor-based *in vitro, ex vivo* and *in vivo* models, on the mRNA and protein level. Based on these results, a combined entinostat plus EGFR inhibitor erlotinib treatment *in vitro* and *in vivo* was studied.

**Results:**

Proteomics analyses in gastric cancer cells treated with entinostat revealed a marked upregulation of EGFR in the majority of cell lines and an even more robust induction of the EGFR ligand AREG. This was confirmed in a panel of different cell lines *in vitro*, in tumor tissue-slice cultures *ex vivo* and in cell line- or patient-derived tumor xenografts in mice. Since previous studies in other tumor entities showed a downregulation of EGFR by HDACi, our findings thus indicate essential differences in the adaptive response of gastric carcinoma cells. Moreover, our results provided the basis for combined entinostat + EGFR inhibitor (erlotinib) treatment, and indeed we demonstrate synergistic effects in combination therapy studies.

**Conclusion:**

Our findings establish the profound upregulation of the EGFR/AREG axis by entinostat as starting point for a rational combination therapy in gastric carcinoma.

## Introduction

In recent years, it has become evident that epigenetic alterations play a significant role in gastric cancer [1]. The different levels of epigenetic changes include the dysregulation of distinct microRNAs, deviations in the DNA methylation pattern in critical genes and modifications of histone proteins, for example through the altered activity of histone deacetylases (HDAC). Interestingly, class I HDACs, in particular the HDAC subtypes HDAC1 and HDAC2, were found to be upregulated in gastric carcinoma in a number of studies and there are indications of a correlation with the prognosis of this disease [2].

Owing to the importance of epigenetic alterations in gastric carcinoma, modulators of epigenetic processes (so-called ‘epidrugs’) have been explored in gastric carcinoma in the preclinical setting. Of note, inhibitors of histone deacetylases (HDACi) showed promising activity against gastric cancer cells, especially in combination with standard chemotherapeutic agents or irradiation [[3], [4], [5], [6], [7], [8], [9]]. Unfortunately, these positive preclinical findings could not yet be reproduced in the relatively few clinical studies in patients [[10], [11], [12]]. This indicates that more suitable and synergistically effective combination partners for HDACi need to be identified, representing an approach that is currently being pursued in other solid tumors as well [13]. Rational combination therapies could be derived from adaptive molecular responses of HDACi-treated tumor cells, which contribute to resistance to these inhibitors.

In pursuing this concept, we analyzed for the first time the effects of a class I-selective HDACi treatment on the possible upregulation of components of the oncogenic EGF receptor (EGFR) signalling pathway (receptor or ligands) in gastric cancer cells. This hypothesis-driven approach was based on data emerging from proteome analyses of entinostat-treated gastric cancer cells, unexpectedly demonstrating the upregulation of EGFR and the EGFR ligand amphiregulin in cells exposed to this HDACi. While this may well lead to decreased antitumor efficacy of HDACi, it can simultaneously provide the basis for increased efficacy upon combined inhibition of both, HDAC activity and EGFR signalling. Remarkably, for other tumor entities mainly inhibitory effects of HDACi on EGFR expression have been described, and their influence on ligands of EGFR is generally under-investigated so far.

Therefore, entinostat effects on the expression of EGFR and amphiregulin were studied in a panel of gastric cancer cell lines, tumor slice cultures and patient-derived xenografts. This included tumor models without basal overexpression of EGFR and thus without primary susceptibility towards EGFR inhibition, as well as cell lines with genomic HER2 or MET amplification, for testing whether constitutive activation of other, RTK-dependent signalling pathways may represent a principle obstacle to the induction of acquired vulnerability to EGFR inhibitors. Based on our findings, we also tested the combination of entinostat and the EGFR inhibitor erlotinib in cell culture and in murine tumor xenografts.

## Material und methods

### HDAC inhibitors

For the inhibition of HDACs the clinically approved class I-selective inhibitor entinostat (MS-275) was mainly used in this study. As additional class I HDACi the experimental agent VK1 [14] and the clinically approved inhibitor romidepsin (FK 228) were included in some experiments. Furthermore, the unselective “pan”-HDACi vorinostat (SAHA) was employed. Entinostat, romidepsin, and vorinostat were from MedChemExpress (Monmouth, USA). VK1 was a generous gift from Finn K. Hansen and Linda Schäker-Hübner (Pharmaceutical Institute, University Bonn, Germany).

### Cell culture and treatment

The human gastric cancer cell lines MKN-7 (RRID: CVCL_1417), MKN-45 (RRID: CVCL_0434), MKN-74 (RRID: CVCL_2791) and NCI-N87 (RRID: CVCL_1603) were purchased from the American Type Culture Collection (ATCC, Manassas, VA, USA). GES-1 cells (RRID: CVCL_EQ22) are SV40 transformed normal gastric epithelial cells [15] and were also purchased from ATCC (Manassas, VA, USA). FEF3 cells are primary human fibroblasts derived from fetal esophagus [16,17]. FEF3 cells were cultured in DMEM medium (Thermo Fisher, Darmstadt, Germany) supplemented with 10 % fetal calf serum (SERANA, Pessin, Germany). All other cells were cultured in RPMI-1640 with phenol red and sodium pyruvate (Sigma Aldrich, Taufkirchen, Germany) supplemented with 10 % fetal calf serum (SERANA, Pessin, Germany) and 300 mg/l glutamine. All cells were grown under standard conditions (37°C, 5 % CO_2_) in a humidity-controlled incubator. Cell lines were authenticated by short repeat tandem profiling within the last 3 years and were cultured for <15 passages. All cell line cultures were regularly analyzed for mycoplasma contamination using a PCR mycoplasma detection kit (Venor GeM Classic, Minerva Biolabs, Berlin, Germany). Treatment of cells *in vitro* was conducted 24 h after seeding. Vehicle-treated cells (DMSO) were utilized as the negative control.

### RNA isolation and RT-qPCR

RNA isolation and RT-qPCR procedures were conducted according to standard protocols using phenol / guanidine isothiocyanate for RNA isolation and the RevertAid RT Kit (Thermo Fisher Scientific, Waltham, MA, USA) and PerfeCTa® SYBR® Green FastMix® ROX (QuantaBio, Hilden, Germany) for RT-qPCR. The procedures are described more in detail in the supplemental methods section (see Suppl. Methods).

### Protein analyses by immune blot

For analyses of protein expression by immune blot, whole cell lysates in RIPA buffer (20 mM Tris-HCl, pH 7.5, 150 mM NaCl, 1 % NP-40, 1 % sodium deoxycholate) were used. Proteins were then separated by denaturing 10 % SDS-polyacrylamide gel electrophoresis, prior to transfer of the protein bands onto a 0.2 µm nitrocellulose membrane using electroblotting. Antibodies used for immune blots are described in Suppl. Table 2. More details are given in the supplemental methods section (see Suppl. Methods).

### ELISA

The tissue slices of the patient material were cultivated in air liquid interface culture as described below, with entinostat or DMSO added to the medium. After 48 h / 72 h, the slices were lysed in 30 µl RIPA buffer and the protein concentration was determined using the BCA protein assay. The AREG ELISA (R&D systems) was then performed as described in the manufacturer's manual. The absorbance of each well was measured using an ELISA plate reader (Multiskan FC, Thermo Scientific, Darmstadt, Germany).

### Immunohistochemistry

Immunohistochemistry was performed using sections from paraffin-embedded tumor specimen. From paraffin-embedded tumors, 5 µm thick sections were prepared. The slides were incubated overnight at 4°C with the primary antibody, followed by washing in PBST and incubating for 2 h with the secondary antibody, diluted 1:500 in PBST. After another washing step, the slides were mounted with DAPI mounting medium (ThermoFisher) and dried for 24 h. Further details are provided in the Suppl. Methods section and in the Suppl. Table 2.

### Flow cytometry

Depending on the cell line, the cells were seeded with a cell density of 0.35 to 1.5 × 10⁵ cells/well in a 12-well. The next day, cells were treated with the appropriate entinostat concentration. DMSO was included as a negative control, corresponding to the highest entinostat concentration. After 72 h of treatment, the cells were detached from the 12-well plates using trypsin and fixed in 4 % formaldehyde for 15 min. The cells were then washed 3 times with PBS (1x) and non-specific binding was blocked using 5 % BSA in PBS for 30 min at RT. After another washing step, the primary antibody (anti-EGFR, ThermoFisher), diluted 1:100 in FACS buffer (0.5 % (w/v) BSA and 0.1 % (w/v) NaN_3_ in PBS) was added to the cells, followed by an overnight incubation at 4°C. On the following day, the cells were washed again, the secondary antibody (Alexa Fluor® 647 coupled anti-mouse IgG, Cell Signaling), diluted 1:1000 in FACS-Puffer, was added to the cells and incubated for one hour at RT. Detection was performed after a further washing step using an Attune® Acoustic Focusing Cytometer (ThermoFisher Scientific) in the RL1 channel (650-670 nm).

### Cell counting Kit 8 (CCK8)

Cells were seeded in 96-well plates at a density ranging from 1 to 5 × 10³ cells per well, dependent on the cell line, and were incubated overnight prior to treatment start (day 0). The viability of the cells was assessed using the CCK8 cell counting kit (Dojindo, Munich, Germany) according to the manufacturer's instructions. Briefly, the culture medium was aspirated, and 50 µl of CCK8 reagent, diluted 1:10 in medium without FCS, was added to each well. Following a 1-hour incubation period, the absorbance at 450 nm was measured using an ELISA plate reader (Multiskan FC, Thermo Scientific, Darmstadt, Germany). To correct for background signal, the absorbance was also measured in a well without cells, and this value was subtracted from the other measurements.

### Colony forming assay

To evaluate clonogenic growth 1 × 10⁵ cells / well were seeded into a 6-well plate. Cells were treated with the respective agents for 72 h. Afterwards, cells were trypsinized and 1000 cells per sample were re-seeded in 6-wells without further treatment. After 14 days of growth, with a medium change when appropriate, the colonies were stained with methylene blue (1 mg/ml in 50 % (v/v) ethanol) and counted using ImageJ (NIH, Bethesda, MD, USA).

### Establishment of subcutaneous tumor xenograft models (patient-derived xenografts (PDX) and cell line-based xenografts)

Immunodeficient NOD/SCID/IL2r gamma (null) mice were housed in a controlled environment (23°C, constant humidity) with a 12-hour light/dark cycle and provided with species-specific food and water *ad libitum*. All animal studies were conducted in accordance with national regulations and were approved by local authorities (Landesdirektion Sachsen). The experiments adhered to the ARRIVE guidelines and complied with the EU Directive 2010/63/EU for animal experiments.

Gastric cancer patient-derived xenografts (PDX) were established from surgical material from two patients (see Suppl. Table 3). Approval from the local ethics committee (Faculty of Medicine at Leipzig University) has been obtained (Project number LMB-UCCL-2020_02) and patients who donated tissue had given their written and informed consent. The freshly resected surgical material was first examined as part of the clinical-pathological diagnostics. Only surplus material that was no longer required for further diagnostic procedures was then used for the scientific experiments in this project. To ensure the anonymity of the donors, the samples were used in pseudonymized form.

The implantation of the gastric adenocarcinoma tumors followed a protocol described previously [18]. Briefly, mice were administered metamizol intraperitoneally, followed by isoflurane anesthesia. A tumor specimen of 30 mm³ was implanted into a subcutaneous pouch in the flank region of the mice, and the wound was closed using histoacryl tissue adhesive. The time between surgical removal of the tumor tissue and primary engraftment into the mice did not exceed 4 h. To prevent wound infection, mice received cotrimoxazole (1.45 mg/ml) orally via drinking water for 10 days post-transplantation. PDX tumor tissues were propagated in mice for at least three rounds.

Xenografts from cell lines were established by subcutaneously injecting 5 × 10⁶ cells (MKN-74) suspended in 150 µl PBS into the flanks of the mice.

### *In vivo* therapy studies

For studying therapeutic *in vivo* effects of entinostat, xenograft-bearing mice were randomized into four groups (8-12 animals each) when the tumors reached a size of approximately 80 mm³.

Mice were treated for one week or one month, dependent on the experiment, with entinostat (1 mg/kg body weight), erlotinib (70 mg/kg body weight) or a combination of both, by intraperitoneal injection every third day. Upon termination of the experiment, xenograft tissues were surgically removed for further processing, including RT-qPCR, Western blotting and immunohistochemistry.

### *Ex vivo* tissue slice studies

Using a vibratome as described previously [18], tissue slices of 350 µm thickness were prepared from the explanted tumor xenografts after reaching a size of 6-8 mm in diameter or from primary tumor material from patients. Approval from the local ethics committee (Faculty of Medicine at Leipzig University) has been obtained (Project number LMB-UCCL-2020_02) and patients who donated tissue had given their written and informed consent. Tissue slice pieces of equal size, excluding necrotic tumor areas, were obtained using a 3 mm biopsy punch and transferred onto cell culture inserts for air-liquid interface culture. After 24 h, treatment of these tumor pieces was started by replacing the medium in the bottom compartment with 1 mL medium containing 1.0 - 5.0 µM entinostat or DMSO (1:4000) as vehicle control. Tissue slice punches were harvested after 72 h for RNA isolation or after 96 h for the preparation of protein lysates or immunohistochemistry.

### Proteomics analyses

The starting point of this investigation were proteomics analyses in gastric cancer cells, which are detailed in [19]. The whole data-set is deposited to ProteomeXchange Consortium via the PRIDE partner repository, with the dataset identifier PXD050328. Briefly, gastric cancer cells were treated for 72 h with vehicle DMSO or 3 µM entinostat (three independent samples) and cell lysates were analyzed in a single-run label-free MS-based proteomics approach using dia-PASEF (Suppl. Fig. 1). From 97,278 quantified peptides in MKN74 samples, 8,821 and 10,029 protein groups were inferred.

### Statistics

All *in vitro* / *ex vivo* experiments were independently conducted at least three times. Western blot data were analyzed using ImageJ software (NIH, Bethesda, MD, USA). Statistical significance was determined by performing t-test using SigmaPlot14, with *, *p* < 0.05; **, *p* < 0.03; ***, *p* < 0.01.

## Results

### Entinostat treatment affects EGFR expression in gastric cancer cells

The present study was inspired by proteomics analyses of our group in gastric cancer cells [19]. In this study, cells stimulated with 3 µM entinostat were compared with cells treated with vehicle (DMSO) (Suppl. Fig. 1A). For the cell line MKN-74, 5,309 significantly regulated proteins were obtained by applying a permutation-based threshold for false positive hits of 0.05. Of note, in a re-evaluation of these data we found that 110 proteins related to receptor tyrosine kinase signalling were differentially regulated by entinostat treatment in MKN-74 cells (Suppl. Fig. 1B).

**Fig. 1:**
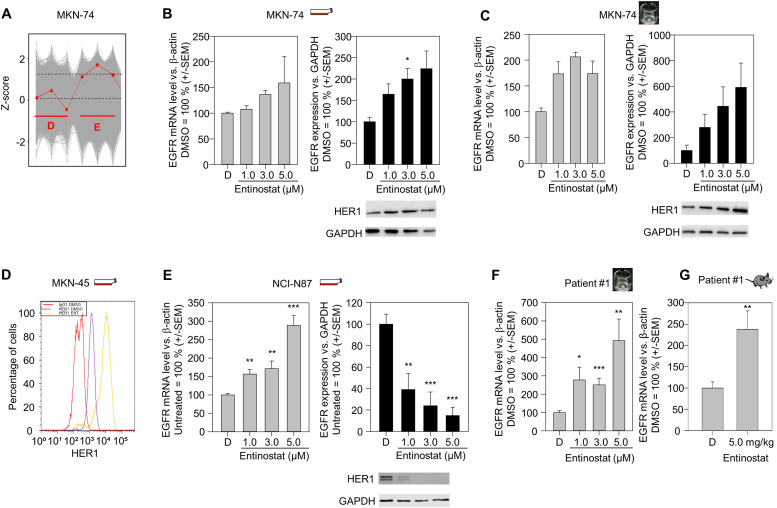
Entinostat effects on EGFR expression in different gastric carcinoma models. (A) Proteomic analyses of the Z-score based deviation of EGFR expression in MKN-74 cells (red line) compared to other evaluated proteins (grey background). The left three dots correspond to EGFR expression under vehicle control (DMSO), while the right three dots correspond to EGFR expression after incubation with 3 µM entinostat for 72 h. Three independent samples of DMSO- and entinostat-treated cells were analyzed. (B, C, E) Determination of EGFR expression on the mRNA level by RT-qPCR (left panels, grey bars) and on the protein level by immunoblot (right panels, black bars) after treatment with increasing concentrations of entinostat vs. vehicle control (DMSO="D") for 48 h. Black bars represent the densitometric quantitation of the EGFR bands in comparison to the loading control (GAPDH) from all experiments, with one representative Western blot shown in each case. In all bar diagrams, the value for DMSO-treated cells is set to 100 %. Results from MKN-74 cells in 2D culture (B), MKN-74 xenograft-derived tissues slices (C) and NCI-N87 cells (E) are shown. (D) FACS-based analysis of EGFR protein expression in MKN-45 cells after treatment with DMSO (purple line) or 3 µM entinostat for 72 h (yellow line). Permeabilized MKN-45 cells were examined. The binding of the EGFR-specific antibody (purple and yellow line) is shown in comparison to an isotype control with a non-specific antibody (red line). (F) EGFR mRNA upregulation in patient #1 PDX-derived tissue slice cultures. (G) Determination of EGFR mRNA expression by RT-qPCR in tumor samples from patient #1 PDX tumor-bearing mice, after daily *in vivo* application of entinostat by intraperitoneal injection for one week. Means + *S*.E.M. are given.

Thus, we further screened alterations in protein expression levels upon 3 µM entinostat treatment of MKN-74 cells. Proteomics analyses revealed that the oncogenic receptor tyrosine kinase EGFR was markedly upregulated in MKN-74 cells (Fig. 1A). This was confirmed in the same cell line on the mRNA level by RT-qPCR and on the protein level by immunoblot (Fig. 1B). However, at the highest entinostat concentration of 5 µM used, there was a reproducible decrease in the GAPDH bands, which is presumably due to the toxic effects of entinostat occurring at this concentration and which was evident here despite the application of the same protein quantities for all samples. In this respect, the increase in EGFR expression in relation to GAPDH should be interpreted with some caution in this case.

Since classical two-dimensional (2D) cell culture with cells growing adherently on a plastic surface resembles only in part the *in vivo* situation, we also analyzed effects in an intact three-dimensional (3D) tumor tissue environment. Treatment of these ∼350 µm thick tissue slice cultures with 5 µM entinostat revealed again a marked upregulation of EGFR mRNA and protein by ∼ 2 and ∼ 6-fold, respectively, which was thus even more profound than in 2D cell culture (Fig. 1C). These data were confirmed in the cell line MKN-45 on the protein level by flow cytometry (Fig. 1D). In contrast, we observed an increase in EGFR mRNA levels but a decrease in EGFR protein levels in 2D cultures of NCI-N87 cells, emphasizing the heterogeneity within gastric cancer cell lines (Fig. 1E).

To get even closer to the patient's situation, we established patient-derived xenografts originating from primary tumors from a moderately differentiated gastric adenocarcinoma (patient #1). Microscopic analyses revealed the preservation of the histology of the primary tumor even after several rounds of propagation in mice (data not shown). In patient #1 PDX tissue slice cultures, treatment with 1 µM entinostat led to a ∼ 3-fold and 5 µM entinostat to a 5-fold increase of EGFR expression, respectively (Fig. 1F). Finally, to study entinostat effects in an *in vivo* situation, PDX-bearing mice (patient #1) were repetitively treated with 5 mg/kg entinostat, every other day for a period of one week. Upon termination of the experiment, mice were sacrificed and the explanted tumors were analyzed for EGFR expression. A ∼ 2.5 upregulation of EGFR was found already after this comparatively short treatment time (Fig. 1G). Taken together, this demonstrates profound effects of entinostat treatment on EGFR receptor expression levels *in vitro* (2D cell culture), *ex vivo* (tissue slice culture) and *in vivo* (xenograft and PDX models).

To exclude that the observed effects were only a substance-specific effect of entinostat we also included analyses with the broad-spectrum HDACi vorinostat and the inhibitors of class I HDACs VK1 and romidepsin. In fact, these structurally unrelated HDACi consistently showed a clear EGFR induction in both cell lines MKN-74 and MKN-45 (Suppl. Fig 2A-C). With this regard the considerably higher potency of romidepsin, acting in the nanomolar range, (Suppl. Fig. 2C) in comparison to vorinostat (Suppl. Fig. 2A) and VK1 (Suppl. Fig. 2B), acting in the micromolar range, was striking. This is in line with its markedly stronger affinity for the class I HDAC subtypes HDAC1 and HDAC2 as compared with the other HDACi. In summary, these findings corroborate that inhibition of class I HDACs HDAC1 and HDAC2 is necessary and sufficient to modulate EGFR expression.

### The EGFR ligand amphiregulin is upregulated under entinostat treatment

**Fig. 2:**
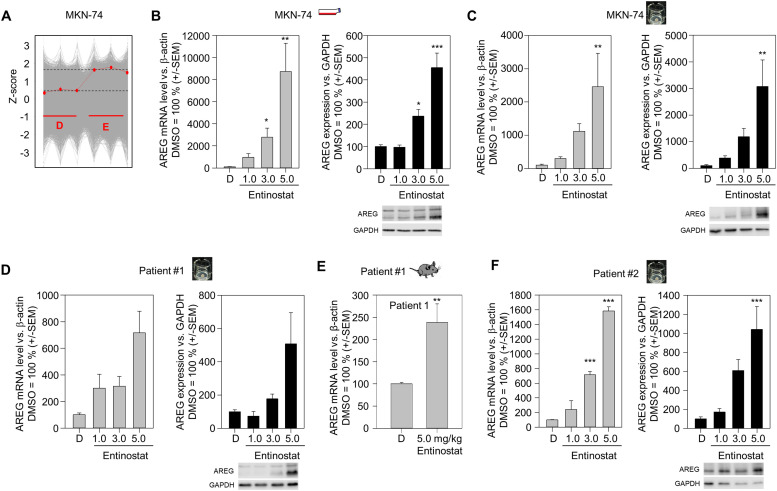
Entinostat mediates the upregulation amphiregulin expression in different gastric carcinoma models. (A) Proteomic analyses of the Z-score based deviation of amphiregulin expression in MKN-74 cells (red line) compared to other evaluated proteins (grey background). The left three dots correspond to amphiregulin expression under vehicle control (DMSO), while the right three dots correspond to amphiregulin expression after incubation with 3 µM entinostat for 72 h. Three independent samples of DMSO- and entinostat-treated cells were analyzed. (B, C) Determination of amphiregulin expression in MKN-74 cells on the mRNA level by RT-qPCR (left panels, grey bars) and on the protein level by immunoblot (right panels, black bars) after treatment with increasing concentrations of entinostat vs. vehicle control (DMSO="D") for 48 h. Black bars represent the densitometric quantitation of the amphiregulin bands in comparison to the loading control (GAPDH) from all experiments, with one representative Western blot shown in each case. In all bar diagrams, the value for DMSO-treated cells is set to 100 %. (D, E) Amphiregulin upregulation (mRNA, left panels, and protein, right panels) in patient #1 (D) and patient #2 (E) PDX-derived tissue slice cultures. (F) Determination of amphiregulin mRNA expression by RT-qPCR in tumor samples from patient #1 PDX tumor-bearing mice, after daily *in vivo* application of entinostat by intraperitoneal injection for one week. Means + *S*.E.M. are given.

Amphiregulin (AREG) is a member of the epidermal growth factor family, acting as an agonist for EGFR. Notably, proteomics analyses of MKN-74 cells after entinostat treatment revealed a profound upregulation of AREG (Fig. 2A). This effect was again found to be dose-dependent in MKN-74 cells on the mRNA- (Fig. 2B, left) and the protein level (Fig. 2B, right). Comparable to EGFR, an even more profound 20-fold increase of AREG mRNA (Fig. 2C, left) and 30-fold increase of AREG protein levels was observed in MKN-74 tumor xenograft tissue slice cultures (Fig. 2C, right). Results on AREG upregulation were also confirmed in MKN-45, MKN-7 and NCI-N87 cells (Suppl. Figs. 3A-C) in 2D culture, as well as in NCI-N87-based tissue slices (Suppl. Fig. 3D).

In line with the above findings on EGFR expression upon entinostat treatment, AREG was found increased in tissue slices from patient #1 PDX as well (Fig. 2D). Interestingly, tissue slices derived from patient #2 PDX tumors revealed an even more profound, entinostat dose-dependent upregulation of AREG (Fig. 2E), despite little entinostat effects on EGFR (data not shown). This also demonstrated that the entinostat-mediated upregulation of the ligand (AREG) is not necessarily paralleled by the increased expression of its receptor (EGFR). In contrast, the *in vivo* treatment of patient #1 PDX tumor-bearing mice confirmed the parallel ∼ 2.5-fold upregulation of EGFR (see Fig. 1G) and AREG (Fig. 2F).

In the next step, the effects the class I-selective HDACi VK1 (Suppl. Fig. 4A) and romidepsin (Suppl. Fig. 4B) on AREG expression were tested. It was found that both HDACi also induced AREG in both MKN-74 and MKN-45 cells (Suppl. Fig. 4A,B). Of note, MKN-74 cells showed a higher AREG induction upon treatment with these agents than MKN-45 cells, which paralleled the effects in these two cell lines upon entinostat treatment (Fig. 2B, Suppl. Fig. 2A). Overall, the comparable effects of romidepsin and VK1 with entinostat indicate that AREG upregulation is not a compound-specific effect of entinostat, but rather a group effect of class I-selective HDACi.

In the proteomics analysis (Suppl. Fig. 1, Fig. 1A and Fig. 2B), AREG was the only EGFR ligand that was upregulated by entinostat. Nevertheless, we performed additional analyses of basal and entinostat-dependent mRNA expression of EGF, HB-EGF and TGF-α in order to gain an impression of whether parallel effects to AREG induction could be observed (Suppl. Fig. 5). Interestingly, very profound differences in the ligands were found at the basal expression level. Whereas for EGF the basal expression levels were close to the detection limit in all cell lines (Suppl. Fig. 5A-C), TGF-α showed very high expression in all gastric carcinoma cells examined (Suppl. Fig. 5A-C). HB-EGF expression was higher expressed than AREG in two of the three cell lines (Suppl. Fig. 5A, C) and was in the range of AREG expression in NCI-N87 cells (Suppl. Fig. 5B).

Interestingly, there were also differential effects on the ligands after entinostat treatment. For EGF, a rather weak effect of entinostat was detected in MKN-74 cells (Suppl. Fig. 5D). In contrast, a massive induction occurred in MKN-45 and NCI-N87 cells (Suppl. Fig. 5E,F), although this was primarily caused by the very low basal levels. In the case of HB-EGF, a more uniform upregulation occurred for all three cell lines (Suppl. Fig. 5G-I). For TGF-α no induction at all occurred in MKN-74 cells (Suppl. Fig. 5 J), a rather small upregulation was found in the cell line NCI-N87 (Suppl. Fig. 5 K), and a strong induction could be demonstrated in MKN-45 cells (Suppl. Fig. 5 L). Although the proteomics data suggest that in the case of the investigated cell lines, AREG is the predominant ligand regulated by HDACi on the protein level, a more detailed analysis of other EGFR ligands in different cell lines both on protein and mRNA level may reveal that EGFR stimulation upon HDACi can occur via different ligands in parallel.

Moreover, we tested the effect of HDACi treatment in non-malignant cells, namely in GES-1 cells as a model for normal gastric epithelium and additionally in the fibroblast cell line FEF3, as a model for stromal cells in a tumor [17]. For EGFR an approximately 1.5-fold upregulation by the highest entinostat concentration of 5 µM occurred in FEF3 cells (Suppl. Fig. 6A, left), whereas no upregulation occurred in the epithelial cell line (Suppl. Fig. 6A, right). With respect to AREG there was already a marked difference in the basal expression levels in both non-malignant cells as compared to the tumor cell lines with untreated (data not shown) or vehicle-treated (Suppl. Fig. 6B) GES-1 and FEF3 cells expressing AREG in the range of the detection limit. Of note, the fibroblast FEF3 cell line then showed a substantial induction of AREG after entinostat treatment with expression levels almost comparable to the tumor cells (Suppl. Fig. 6B). In contrast, epithelial GES-1 cells only showed a minor upregulation of AREG upon entinostat treatment (Suppl. Fig. 6B). In this respect, the effects of HDACi on the AREG-EGFR axis in gastric carcinoma cells do not appear to be an indication of a general mechanism that is also established in non-malignant gastric epithelial cells.

Since EGFR signal transduction is known to include the MAPK pathway, we next analyzed ERK1/2 activation. In this context, it is important to mention that the treatment of the cells with HDACi was carried out over 72 h, in analogy to the analyses of the protein expression of AREG and EGFR. This means that due to the long duration of treatment, not only the post-translational modification of ERK1/2 (i. e., phosphorylation) may be critical for the activity of this signaling pathway, but also the level of total ERK1/2 protein expression. To assess these complex effects separately, ERK phosphorylation (P-ERK1/2), total ERK (ERK1/2) and GAPDH (as an independent loading control) were evaluated (see the representative blots in Suppl. Fig. 7A-C and the separate quantification against GADPD (Suppl. Fig. 7A-C) and total ERK1/2 (Suppl. Fig. 8A-C).

Indeed, 3 µM entinostat led to an approximately 2-fold increase in ERK1/2 phosphorylation in MKN-74 (Suppl. Fig. 7A left, Suppl. Fig. 8A, left) cells. With an even higher entinostat concentration (5 µM) a plateau was reached with a trend towards lower ERK1/2 phosphorylation (Suppl. Fig. 7A, left, Suppl. Fig. 8A, left). In contrast, a decrease in ERK1/2 phosphorylation occurred in NCI-N87 cells (Suppl Fig. 7B, left) indicating that this cell line was more sensitive towards entinostat inhibitory and cytotoxic effects which may also affect MAPK signalling. Such potentially toxic effects of entinostat in NCI-N87 cells were also indicated by the slight decrease in GAPDH and total ERK1/2 expression, which occurred despite the application of equal amounts of protein. However, since the decrease in ERK1/2 phosphorylation was greater than the reduction in GAPDH and total ERK1/2 bands, a decrease in the P-ERK1/2 signal occurred both in relation to GAPDH (Suppl. Fig. 7B, left) as well as to total ERK1/2 bands (Suppl. Fig. 8B, left). This suggests that interference of entinostat with ERK1/2 phosphorylation was the predominant mechanism for the observed effect. Interestingly, slice cultures of NCI-N87 showed a partial stimulation of ERK1/2 phosphorylation in relation to GAPDH (Suppl. Fig. 7B, right) and a partial inhibition in relation to total ERK1/2 (Suppl. Fig. 8B, right). A differential picture also emerged in slice cultures of PDX, as both ERK1/2 phosphorylation and total ERK1/2 expression were reduced by entinostat relatively parallel to each other, whereas GAPDH bands were less affected by eninostat (Suppl. Fig. 7C). Accordingly, there was a decrease in ERK1/2 phosphorylation compared to GAPDH (Suppl. Fig. 7C) and no clear effect with regard to the P-ERK1/2 to ERK1/2 ratio (Suppl. Fig. 8C). The latter example illustrates that in this complex situation after long-term treatment GAPDH represents a valuable control for ERK1/2 phosphorylation in addition to normal ERK1/2 bands.

### Entinostat-mediated upregulation of EGFR and AREG in primary tumors from patients reveals some inter-tumor heterogeneity

**Fig. 3:**
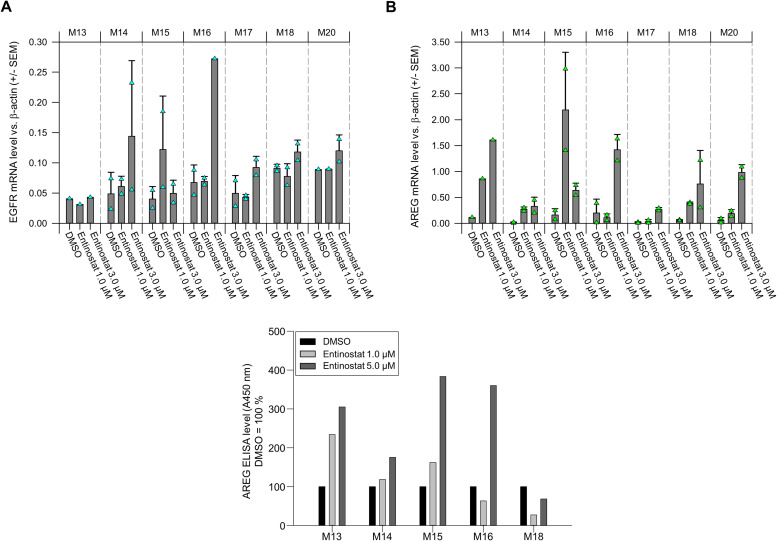
Analysis of EGFR and amphiregulin expression in primary samples of gastric cancer patients grown as tumor slice culture. (A) EGFR and (B) amphiregulin mRNA expression was determined by RT-qPCR upon incubation with vehicle DMSO or entinostat at the given concentration for 72 h. Shown are the mean values of two technical replicates (cyan and green triangles, respectively) of seven patients. (C) Amphiregulin secretion into the supernatant of tumor slice cultures of primary tumors from five patients upon stimulation with vehicle DMSO or entinostat in the given concentrations for 72 h.

Albeit patient-derived xenografts were generated from primary tumors from patients, their stroma is eventually replaced by murine cells during tumor growth and propagation in mice. This may lead to effects not precisely representing the clinical situation of a patient's tumor. In order to address this and to further extend the numbers of tumors analyzed for entinostat effects, we screened a panel of seven primary tumors from different patients in tissue slice ALI culture. On the mRNA level, 5 out of 7 tumors showed an upregulation of EGFR already at 3 µM entinostat. One tumor showed upregulation at low entinostat concentrations with a subsequently inverse dose-dependence, and in only one tumor EGFR levels remained unchanged (Fig. 3A). While the sometimes moderate increases may be accounted to the entinostat concentration being lower here (3 µM) as compared to above (up to 5 µM), this was not an issue in the case of AREG. Here, a marked upregulation (sometimes from very low levels) was seen in all primary tumor samples (Fig. 3B). One tumor (M15) showed again an upregulation at low entinostat dosages and a subsequently inverse dose-dependence, with AREG overexpression being more profound upon treatment with 1 µM entinostat as compared to 3 µM. For five tumors selected based on the availability of sufficient tumor material, AREG upregulation was also studied on the protein level. An ELISA for quantitation of AREG in the tissue slice lysates revealed entinostat-mediated increases in AREG protein levels in 4 out of 5 primary tumors (Fig. 3C). Only one tumor showed no alterations in AREG expression.

Taken together, this demonstrates the effects of entinostat also in primary patient tissue; however, our results also highlight some inter-tumor heterogeneity.

### The activation of the EGFR / AREG axis by entinostat provides the basis for its combination with erlotinib

**Fig. 4:**
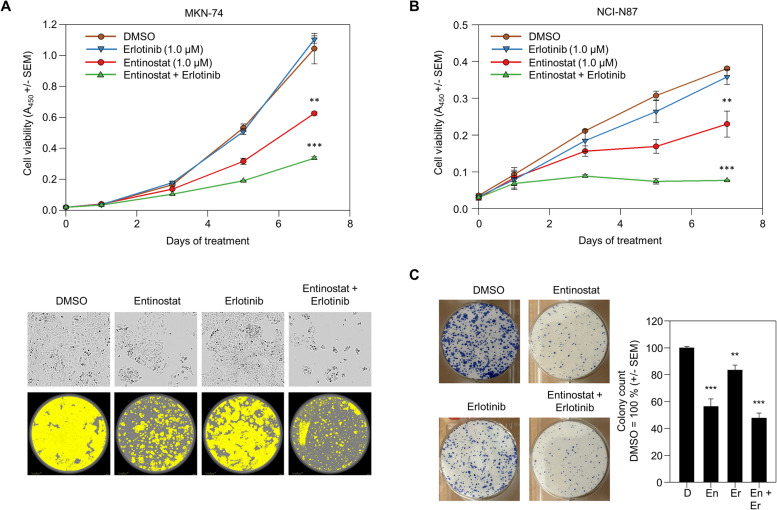
Effect of entinostat and erlotinib single or combination treatment on the proliferation of gastric cancer cells. (A, B) Using the formazan-based WST-8 assay, proliferation of (A) MKN-74 and (B) NCI-N87 cells was evaluated upon treatment with vehicle DMSO, erlotinib, entinostat, or the combination of erlotinib and entinostat. Given are mean values of three independent experiments (± *S*.E.M.). (A, lower panel) Images of the MKN-74 cell proliferation assay, taken at day 5 for evaluating cell confluency. Cell density measurements were performed and quantified using an Incucyte® SX5 (signals shown in yellow in the figure). (C) MKN-74 colony forming assay performed after 48 h of treatment by reseeding 1000 cells per well. The colonies stained with methylene blue are shown in bright field.

The upregulation of EGFR and/or AREG upon entinostat treatment suggests that the tumor cells may become then more sensitive towards EGFR inhibition. We thus employed erlotinib as a clinically approved, selective EGFR inhibitor. At a concentration of 1 µM, erlotinib alone failed to exert any tumor-suppressive effects on MKN-74 cells (Fig. 4A). As seen before, 1 µM entinostat resulted in a moderate cell inhibition. Notably, however, when combining both inhibitors a very profound suppression of tumor cell growth was observed (Fig. 4A). This demonstrated that the otherwise ineffective 1 µM erlotinib treatment became efficient in combination with entinostat. Similar results were obtained in NCI-N87 cells, with very little inhibitory effects of the 1 µM erlotinib single therapy, while the combination with the moderately efficient 1 µM entinostat therapy led to an essentially complete abolishment of tumor cell growth (Fig. 4B). Combined inhibitory effects were also observed in colony formation assays. While in MKN-74 cells profound inhibition of colony formation was already observed under entinostat treatment and, to smaller extent, also in the presence of erlotinib, the combination resulted in a further reduction of colony numbers and sizes (Fig. 4C).

### Synergistic effects entinostat + erlotinib combination therapy *in vivo*

**Fig. 5:**
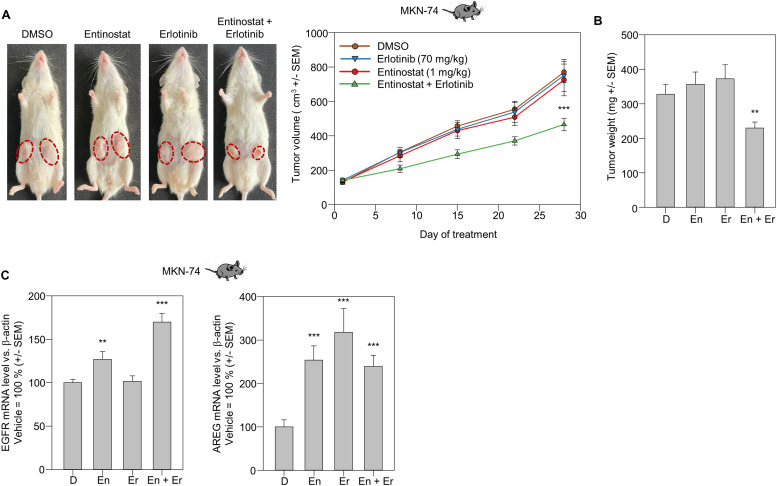
Analysis of combination effects of entinostat and erlotinib treatment *in vivo* in MKN-74 xenograft-bearing mice. (A) Growth of subcutaneously injected MKN-74 tumor cells in mice after systemic treatment (every third day) with the inhibitors (single or combination therapy) as compared to control-treated animals. Mean values of ≥ 8 tumors per treatment group + *S*.E.M. Represantative pictures of mice depicting tumor sizes at day 28 are given. (B) Tumor weight of MKN-74 xenografts at day 28 after control treatment (vehicle DMSO = “D”) or entinostat (En) / erlotinib (Er) single and combination treatment. Mean values of ≥ 8 tumors + *S*.E.M. are given. (C) Determination of EGFR (left) and amphiregulin (AREG) mRNA expression in tumor samples of MKN-74 xenografts at day 28 after the respective treatments (control treatment = “D” vs. entinostat (En) / erlotinib (Er) alone or in combination). Given are mean values + *S*.E.M.

Prompted by the above induction of erlotinib efficacy upon its combination with entinostat and the concomitant overall enhancement of tumor cell inhibition, we treated MKN-74 tumor xenograft-bearing mice with erlotinib and entinostat in a therapy study *in vivo*. At the selected dosages, no anti-tumor effects were seen in the single treatment groups. The combination of both inhibitors, however, led to a significant inhibition of tumor xenograft growth (Fig. 5A). These results from the tumor growth curves were also confirmed by the determination of tumor weights upon termination of the experiment (Fig. 5B).

The analysis of EGFR and AREG mRNA levels in the tumors of the different treatment groups also revealed a slight, but statistically significant upregulation of EGFR in the single therapy group treated with 1 mg/kg entinostat (Fig. 5C, left). In the same group, a more profound ∼ 2.5 increase of AREG mRNA was observed (Fig. 5C, right). Notably, while erlotinib single treatment did not affect EGFR mRNA levels (Fig. 5C, left), a ∼ 3-fold upregulation was found in the case of AREG (Fig. 5C, right). The combination therapy led to an even further increase of EGFR and a still ∼ 2.5-fold upregulation of AREG.

**Fig. 6:**
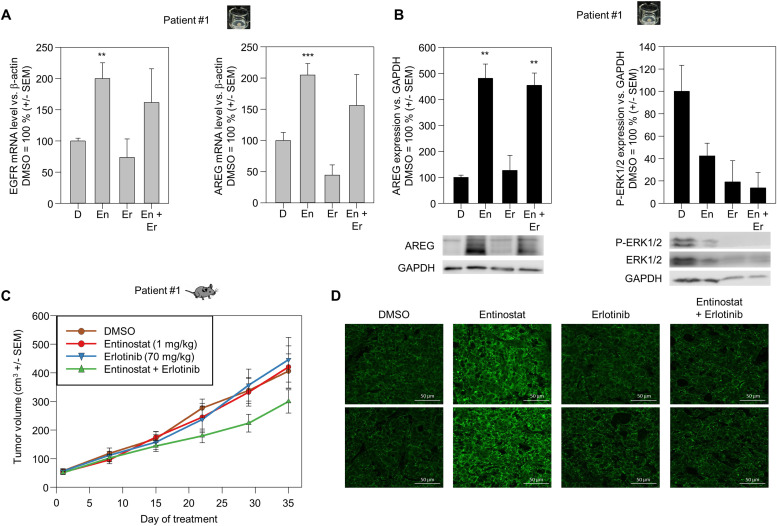
Analysis of combination effects of entinostat and erlotinib treatment *ex vivo* in tumor slice cultures and *in vivo* in PDX tumors (Patient #1) in mice. (A) Determination of EGFR (left) and amphiregulin (AREG) (right) mRNA expression in PDX tumor slice cultures treated *ex vivo* for 72 h (control treatment = “D” vs. entinostat (En) / erlotinib (Er) alone or in combination). Given are mean values of three independent experiments + *S*.E.M. (B) Expression of (left) amphiregulin and phosphorylation (right) of ERK1/2 (P-ERK1/2) as determined by immunoblot in PDX tumor slice cultures treated *ex vivo* for 72 h (control treatment = “D” vs. entinostat (En) / erlotinib (Er) alone or in combination). Shown are densitometric evaluations of the amphiregulin or P-ERK1/2 bands in comparison to the loading control (GAPDH). Values for vehicle treated cells were set = 100 %. Given are mean values of three independent experiments + *S*.E.M. (C) Growth of subcutaneously implanted PDX tumor cells in mice after systemic treatment (every third day) with the inhibitors (single or combination therapy) as compared to control-treated animals. Mean values of ≥ 8 tumors per treatment group + *S*.E.M. (D). AREG expression of the explanted PDX tumors, as determined by immunofluorescence staining (D).

We next switched to patient #1 PDX tumors for further analysis of combination effects on tumor growth and gene expression. Single treatment of PDX tissue slices in ALI culture revealed the previously observed upregulation of EGFR (Fig. 6A, left) and AREG mRNA (Fig. 6A, right). In contrast, erlotinib single treatment led to a slight ∼ 20 % and a more profound ∼ 50 % reduction of EGFR and AREG mRNA levels, respectively. The tumors from the combination treatment group exhibited exactly the combined effects of single treatment, with a partial reversal of the entinostat-mediated increase of EGFR and AREG mRNA (Fig. 6A). The upregulation under entinostat, but not under erlotinib treatment, was also confirmed on the protein level, as shown for AREG (Fig. 6B, left). Notably, however, despite this profound upregulation of both, receptor and ligand, the downstream signal transduction was markedly impaired upon combined inhibitor treatment of the tissue slices, as seen when analysing phospho-ERK1/2 levels as an indicator for MAPK activation (Fig. 6B, right).

When treating PDX-bearing mice *in vivo*, again no tumor-inhibitory effects were observed in both single therapy groups. The combination of the sub-effective dosages of both inhibitors, however, yielded a marked reduction of tumor growth (Fig. 6C). The analysis of the tumors after termination of the experiment by immunofluorescence microscopy revealed the upregulation of EGFR expression upon entinostat treatment (Fig. 6D). Interestingly, in this experimental setting this overexpression was abolished in the combination therapy group, again highlighting the synergistic effects of treatment with both inhibitors.

## Discussion

The effects of entinostat on the amphiregulin-EGFR axis described in this study highlight the complexity and possible issues of HDACi in solid tumors. In addition to the well-established reactivation or induction of tumor suppressor genes such as p53, Rb or PTEN [[20], [21], [22], [23], [24], [25]], which is desirable in the context of tumor therapy, this study demonstrates that HDACi can also lead to an unwanted upregulation or activation of well-established (proto-)oncogenes such as EGFR.

In gastric cancer, molecular effects of HDACi on the expression of EGFR have not been investigated so far. While in other tumor entities HDACi-mediated alterations of EGFR expression appear to be cell context-dependent and may also depend on the HDACi used, findings of EGFR downregulation overall predominate. For example, HDACi have been found to inhibit EGFR expression in non-small cell lung cancer (NSCLC) [[26], [27], [28]], head and neck cancer [[29], [30], [31]], colorectal carcinoma [32], breast cancer [33], pancreatic adenocarcinoma [34] and glioblastoma [35]. Besides these findings relying on broader-spectrum HDACi, decreased EGFR expression was also found after treatment with the HDAC subtype 6 preferential inhibitor WT161 [36]. Differences between HDAC inhibitor types and specificities as well as the fact that the downregulation of EGFR under HDAC inhibition may also depend on the EGFR mutation status add further complexity [26,33]. On the other hand, a few literature findings also describe an EGFR induction under HDACi treatment, e.g. in the NSCLC cell line H520 after treatment with entinostat [37]. Still, entinostat specific properties cannot explain these divergent findings since the same drug was also capable of EGFR downregulation, albeit in a non-tumor context [38]. Conversely, a transient upregulation of EGFR was shown in the prostate carcinoma cell line DU-145 after treatment with the HDACi SN30028, which inhibits not only class I HDACs, but also the class IIb representative HDAC6 [39].

Despite this somewhat heterogeneous picture from the literature, our findings in gastric cancer cells thus mainly differ from those in other entities. It should be mentioned, however, that we found EGFR downregulation in one cell line (NCI-N87) as well, suggesting some heterogeneity even within a given tumor entity. In this context, it should be noted that expression levels of different receptors of the EGF receptor family may also affect each other. As an example from other tumor entities, we were able to demonstrate an adaptive response to EGFR inhibition or HER2 inhibition, leading to a rapid, compensatory counter-upregulation of the respective heterodimerization partner, HER2 or EGFR, respectively [40]. The existence of such adaptive responses could also lead to indirect molecular effects of HDACi, which may even overcompensate their direct effects on the expression of a given HER receptor. Indeed, we could already demonstrate an HDACi-mediated downregulation of HER2 in gastric carcinoma, suggesting that this could at least contribute to a compensatory EGFR upregulation [41].

A more robust and even more pronounced effect of entinostat treatment was observed when switching to the EGFR ligand amphiregulin, with profound upregulation in all gastric carcinoma cell lines. To the best of our knowledge, this is the first study on HDACi effects on an EGFR ligand in gastric cancer. Again, studies in other tumor entities revealed an ambiguous picture, with the HDAC1-, HDAC3- and HDAC6-specific inhibitor SN30028 leading to a persistent downregulation of amphiregulin in the prostate carcinoma cell line PC3 [39]. In contrast, the non-selective HDACi valproic acid enhanced the secretion of amphiregulin in glioblastoma cells, associated with concomitantly increased resistance to temozolomide [42], and in a non-malignant context, HDAC inhibition led to amphiregulin induction in oocytes [43].

The fact that HDACi treatment exerted a predominantly (EGFR) or consistently (AREG) inducing effect in the case of gastric carcinoma cells is striking. However, it seems unlikely that such an important oncogenic axis would be constitutively inhibited in this tumor entity via HDACs, which in turn would be reversed by HDACi treatment. In fact, the gastric cancer cell lines investigated in this study showed substantially higher basal AREG levels than the non-malignant gastric epithelial cell line GES-1. Moreover, HDACi treatment did not upregulate EGFR at all in non-malignant GES-1 cells and led only to a slight induction of AREG in these cells.

Mechanistically, it therefore seems likely that the HDACi-mediated upregulation of the AREG-EGFR axis in gastric carcinoma cells represents a combination of direct and indirect effects. In this context, it should be noted that HDACi not only influence the acetylation of histone proteins but can also induce a corresponding post-translational modification in important other transcriptional regulators like p53 [44], Sp1 [45] or YB-1 [46], which have also been described as critical determinants of AREG [[47], [48], [49]] or EGFR [50,51] expression. Even more complexity is added by the fact that increased acetylation of these transcription factors can lead to either inhibition or activation of their transcriptional activity [52], depending on the target. Since there are also various autostimulatory loops for the expression of components of the EGFR axis [53], it can be assumed that even small initial changes in the expression of components of the EGFR signaling pathway may lead to a self-amplified response. For YB-1, whose post-translational modification, i. e., acetylation, by entinostat has been described in different contexts [54,55], there is indeed evidence for an involvement in a self-stimulatory loop of the AREG - EGFR axis [56]. Accordingly, the above-mentioned mechanisms provide a starting point for further investigating the mechanism of HDACi-mediated upregulation of AREG or EGFR.

Our findings thus offer a promising starting point for further studies into the modulation of the growth factor and HER receptor ligand amphiregulin by HDACi, also bearing in mind its broad relevance in various different tumor entities [57,58]. In this context, it should be noted that EGFR has a significant receptor reserve in many tumors. This means that - especially in the case of overexpression of a non-constitutively active EGFR - only a relatively small proportion of the receptors will need to bind a stimulating ligand in order to generate a significant mitogenic signal [59,60]. Consequently, even in cases where HDACi treatment leads to the downregulation of EGFR to a certain extent, this could be overcompensated by a simultaneous induction of an activating ligand. Taken together, this emphasizes the relevance of growth factors like amphiregulin in adaptive responses and of the precise analysis of alterations of their expression levels. Also, it further highlights the possible role of receptor ligands in patient stratification prior to targeted therapies.

The unwanted entinostat-mediated stimulation of the amphiregulin-EGFR axis in gastric cancer described for the first time in this study may thus be an important factor in an adaptive response of the tumor cells. By counteracting the antineoplastic effects of HDACi, it may be of high relevance particularly in a clinical setting. Indeed, it was shown previously that resistance to entinostat in small cell lung cancer was associated with increased basal expression of EGFR [61]. Remarkably, it was also demonstrated in lung tumor cells that HDAC1 is a kinase substrate of EGFR and that anti-apoptotic effects of HDAC1 can be stimulated via EGFR [62]. This suggests a possible direct link between EGFR-dependent signalling pathways and a function of HDAC1 that could lead to increased therapy resistance in tumors.

The adaptive activation of the EGFR axis in gastric carcinoma cells after HDACi treatment shown in our study not only represents an important resistance factor to HDACi, but also opens up the possibility of combination therapy with EGFR inhibitors in the sense of acquired vulnerability. The concept of acquired vulnerability in general [63] is based on the assumption that treatment with a specific substance leads to an adaptive upregulation of oncogenes resulting in therapeutic resistance to the respective agent. However, this adaptive response may simultaneously lead to a new susceptibility towards inhibitors of the upregulated oncogene.

## Funding Sources

This investigation was funded by the 10.13039/501100001659German Research Foundation (DFG, AI 24/33-1 to A.A.).

## CRediT authorship contribution statement

**Tamara Zenz:** Writing – original draft, Visualization, Methodology, Investigation, Formal analysis, Data curation. **Robert Jenke:** Investigation, Conceptualization. **Denys Oliinyk:** Visualization, Methodology, Investigation, Formal analysis, Data curation. **Sandra Noske:** Investigation. **René Thieme:** Resources, Methodology. **Tim Kahl:** Resources, Methodology. **Ines Gockel:** Resources, Project administration, Methodology. **Florian Meier-Rosar:** Supervision, Methodology, Investigation, Formal analysis, Data curation. **Achim Aigner:** Writing – original draft, Supervision, Project administration, Funding acquisition, Conceptualization. **Thomas RH Büch:** Conceptualization, Investigation, Methodology, Supervision, Writing – original draft.

## Declaration of competing interest

The authors declare that they have no known competing financial interests or personal relationships that could have appeared to influence the work reported in this paper.

## Appendix Supplementary materials

**Suppl. Fig. 1** Proteomics analyses in the MKN-74 cell line after entinostat treatment. (A) Schematic representation of the proteome analysis process with the individual work steps. (B, left) Schematic depiction of up-regulated (red) and down-regulated (blue) proteins after entinostat treatment. Shown are the measured individual values of three independent samples treated either with entinostat (3 µM) or vehicle control DMSO. (B, left and right) 110 proteins related to receptor tyrosine kinase signalling were found to be differentially regulated by entinostat treatment in MKN-74 cells. Image, image 1

**Suppl. Fig. 2** Effect of different HDAC inhibitors on EGFR mRNA level. The cell lines MKN-74 and MKN-45 were treated for 48 h with the pan-HDAC (A) vorinostat or the class I HDAC inhibitors (B) VK1 and (C) romidepsin (Romidepsin) in two different concentrations or DMSO as a vehicle control. Shown are the mean values of three independent experiments + *S*.E.M. Image, image 2

**Suppl. Fig. 3** Dose-dependent upregulation of AREG after entinostat treatment vs. DMSO (D) negative control, as seen on the mRNA- (left panels, grey bars) and protein level (right panels, black bars) in the cell lines (A) MKN-45, (B) MKN-7 and (C) NCI-N87. (D) Results from NCI-N87 cell line-derived xenograft tissue slices. The mean values of three independent experiments + *S*.E.M.) are shown. Image, image 3

**Suppl. Fig. 4** Effect of different HDAC inhibitors on AREG mRNA level. The cell lines MKN-74 and MKN-45 were treated for 48 h with the class I HDAC inhibitors (A) VK1 and (B) romidepsin (Romidepsin) in two different concentration or DMSO as a vehicle control. Shown are the mean values of three independent experiments + *S*.E.M. Image, image 4

**Suppl. Fig. 5** Basal level and impact of entinostat treatment on various EGFR ligands in different gastric cancer cell lines. The basal expression of the EGFR ligands AREG, EGF, HB-EGF and TGF-α on mRNA level (upper A, B and C) was measured by qPCR. Additionally the changes in expression on mRNA level for the ligands EGF, HB-EGF and TGF-α after 48 h of entinostat treatment vs. vehicle control DMSO for the are shown. On display are the mean values of three independent experiments + *S*.E.M. Image, image 5

**Suppl. Fig. 6** Impact of an entinostat treatment on non-neoplastic cell lines. The fibroblast cell line FEF3 and the gastric epithelial cell line GES-1 were treated with entinostat or DMSO as vehicle control for 48 h. Changes of the mRNA-level of (A) EGFR were measured by qPCR compared to beta actin and normalized to an untreated sample. (B) AREG mRNA level of the non-malignant cell lines was normalized to beta actin only and compared to the cancer cell lines MKN-74, MKN45 and NCI-N87. All bars represent the mean of three independent experiments + *S*.E.M. Image, image 6

**Suppl. Fig. 7** Effect of entinostat on EGFR signalling via ERK1/2 phosphorylation. The cell lines (A, left) MKN-74 and (B, left) NCI-N87 were treated for 72 h with entinostat vs. vehicle control (DMSO), prior to analysis of protein samples by immunoblot. The bars represent the quantitation of phosphorylated ERK1/2 bands normalized to the loading control (GAPDH) from three experiments, with DMSO treated cells set to 100 %. Additionally, a representative Western blot is shown in each case. Results from 2D cell culture (A, B, left), tissue slice culture (A, B, right) and patient #2 PDX tissue slices (C) are shown, with bars giving the mean values of three independent experiments + *S*.E.M. Image, image 7

**Suppl. Fig. 8** Quantification of phosphorylated ERK in relation to total ERK after entinostat treatment. The cell lines (A, left) MKN-74 and (B, left) NCI-N87 were treated for 72 h and the tissue slices of (A, right) MKN-74, (B, right) NCI-N87 and (C) PDX material (patient #2) were treated for 72 h with entinostat or the vehicle control DMSO. Changes in expression were detected by Western blot. The mean values of 2-3 independent experiments + *S*.E.M. are shown. Image, image 8

Image, application 9
