# Supplementary figures and images for: Acquired vulnerability against EGF receptor inhibition in gastric cancer promoted by class I histone deacetylase inhibitor entinostat
Source: Neoplasia. 2025 Jan 25;60:101121. doi: 10.1016/j.neo.2024.101121 (PMC11802376; doi:10.1016/j.neo.2024.101121)

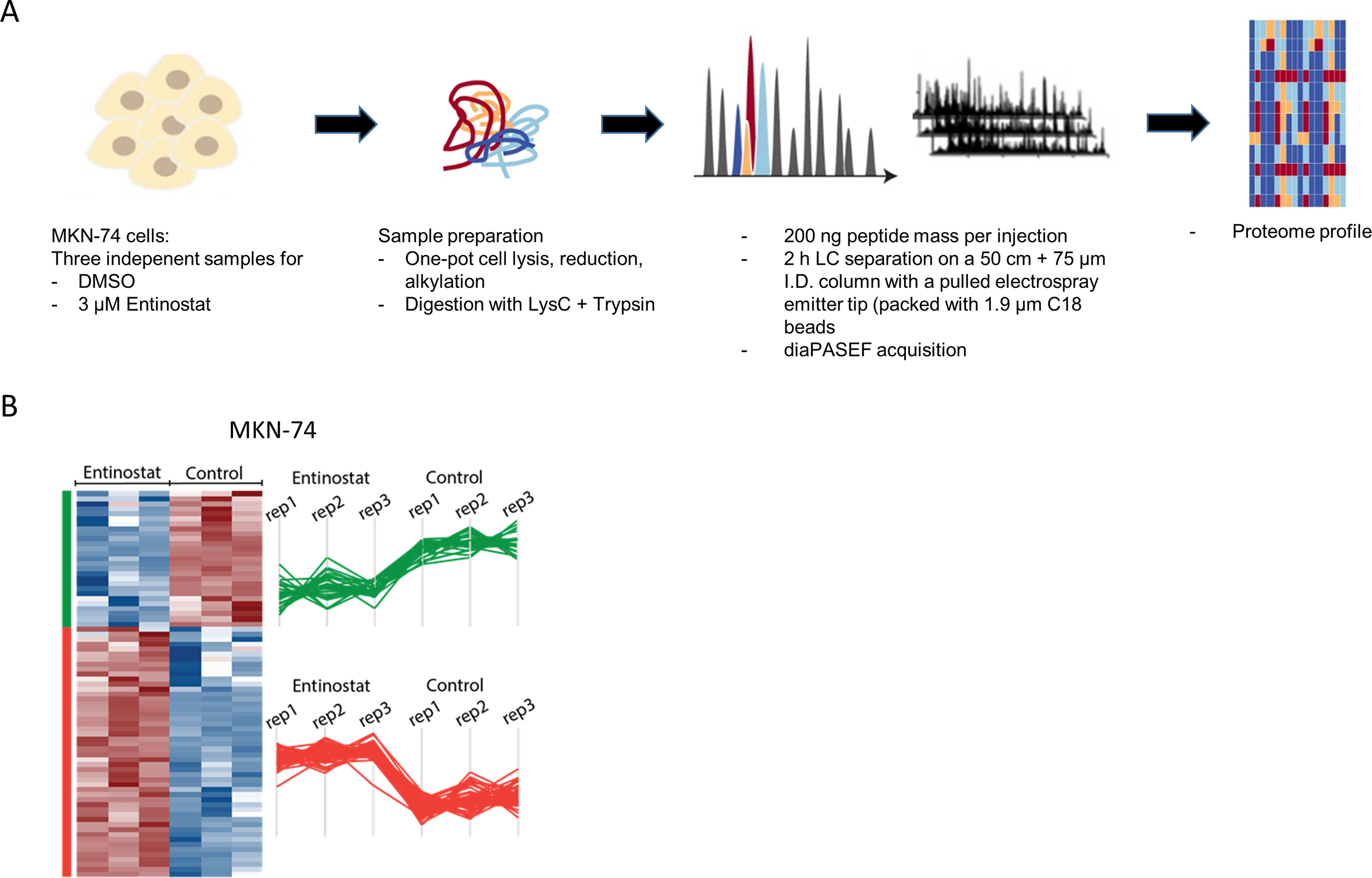

Supplement: Supplementary file 1 — Suppl. Fig. 1 Proteomics analyses in the MKN-74 cell line after entinostat treatment. (A) Schematic representation of the proteome analysis process with the individual work steps. (B, left) Schematic depiction of up-regulated (red) and down-regulated (blue) proteins after entinostat treatment. Shown are the measured individual values of three independent samples treated either with entinostat (3 µM) or vehicle control DMSO. (B, left and right) 110 proteins related to receptor tyrosine kinase signalling were found to be differentially regulated by entinostat treatment in MKN-74 cells. [file mmc1.jpg]

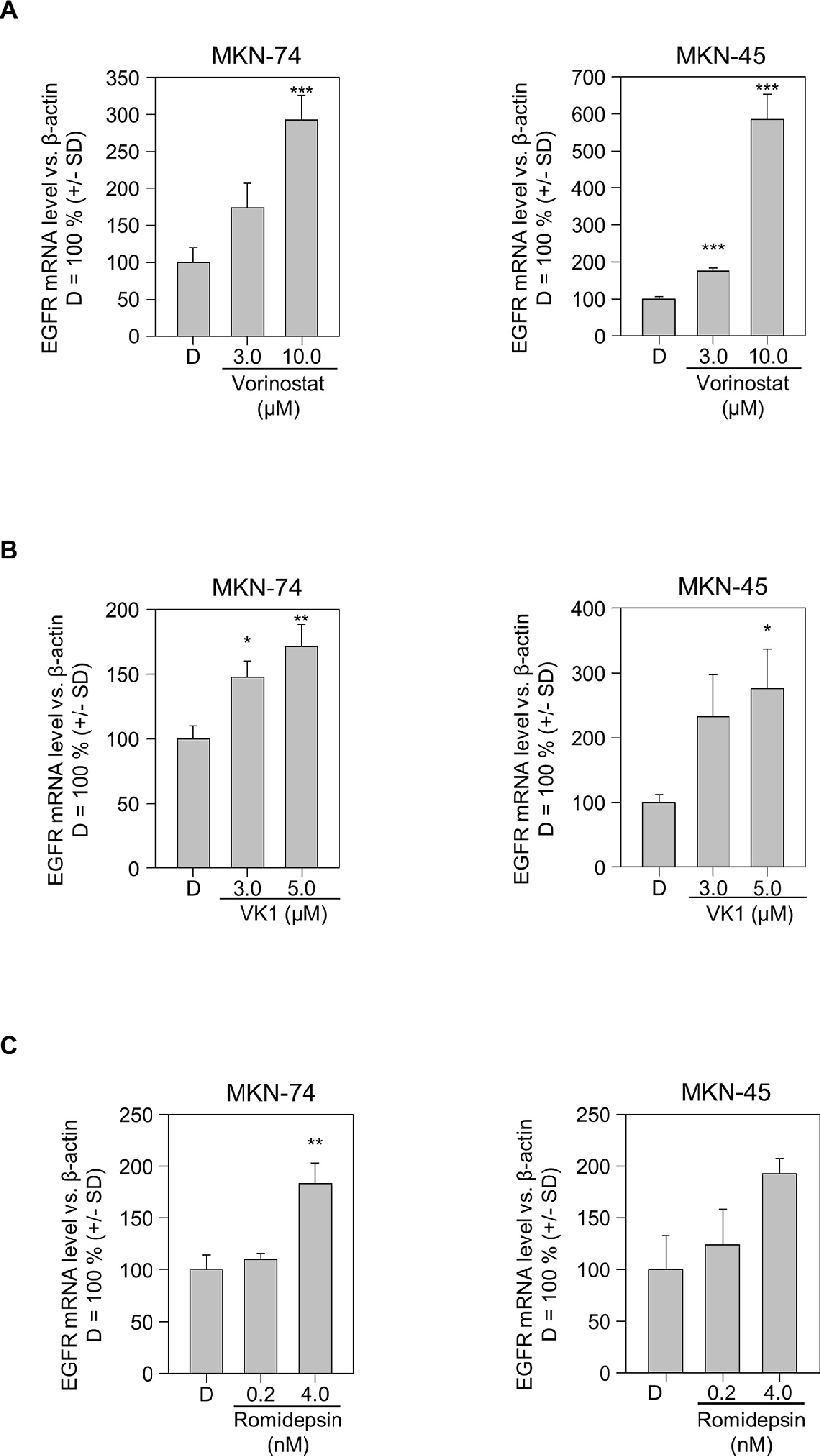

Supplement: Supplementary file 2 — Suppl. Fig. 2 Effect of different HDAC inhibitors on EGFR mRNA level. The cell lines MKN-74 and MKN-45 were treated for 48 h with the pan-HDAC (A) vorinostat or the class I HDAC inhibitors (B) VK1 and (C) romidepsin (Romidepsin) in two different concentrations or DMSO as a vehicle control. Shown are the mean values of three independent experiments + S.E.M. [file mmc2.jpg]

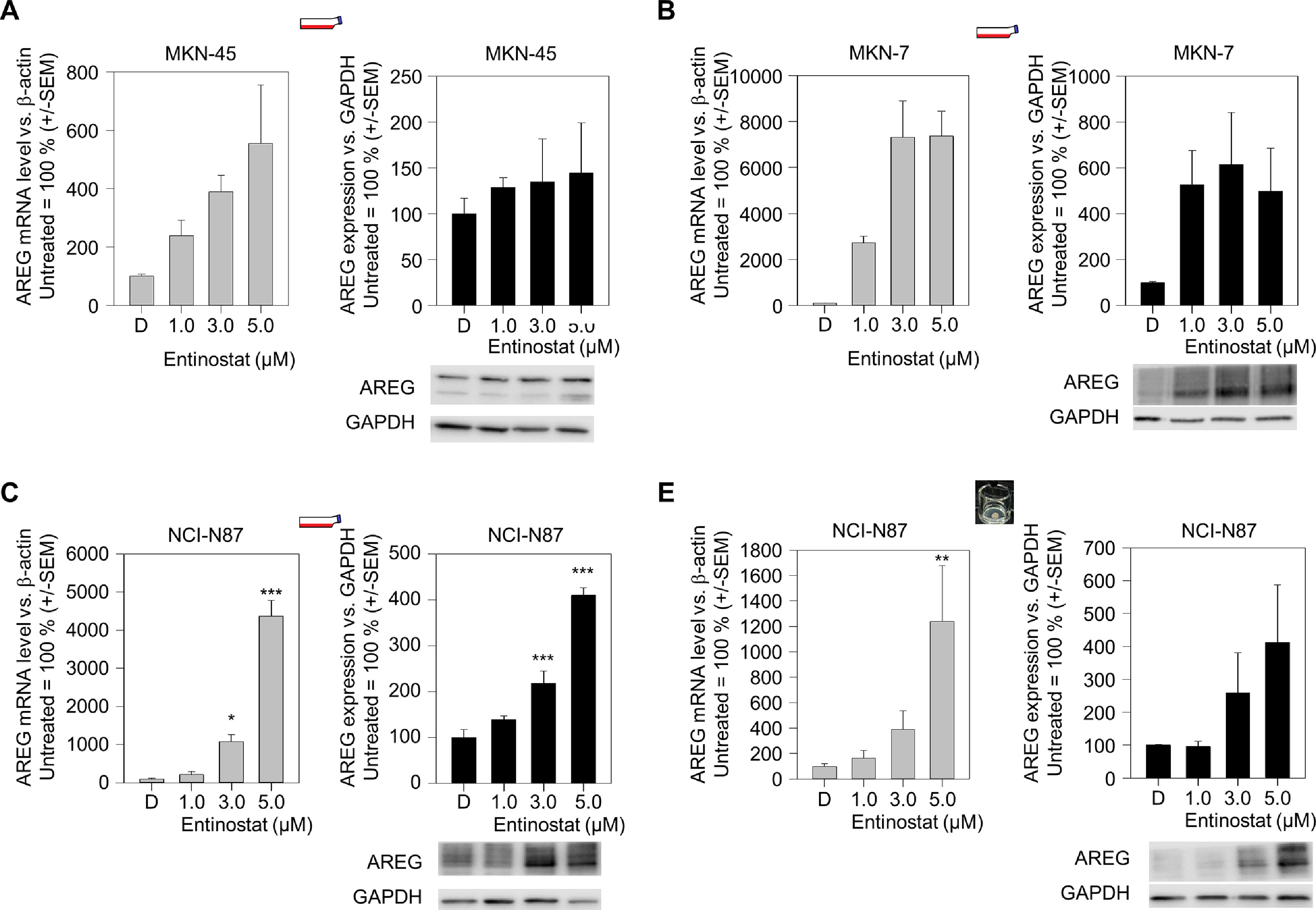

Supplement: Supplementary file 3 — Suppl. Fig. 3 Dose-dependent upregulation of AREG after entinostat treatment vs. DMSO (D) negative control, as seen on the mRNA- (left panels, grey bars) and protein level (right panels, black bars) in the cell lines (A) MKN-45, (B) MKN-7 and (C) NCI-N87. (D) Results from NCI-N87 cell line-derived xenograft tissue slices. The mean values of three independent experiments + S.E.M.) are shown. [file mmc3.jpg]

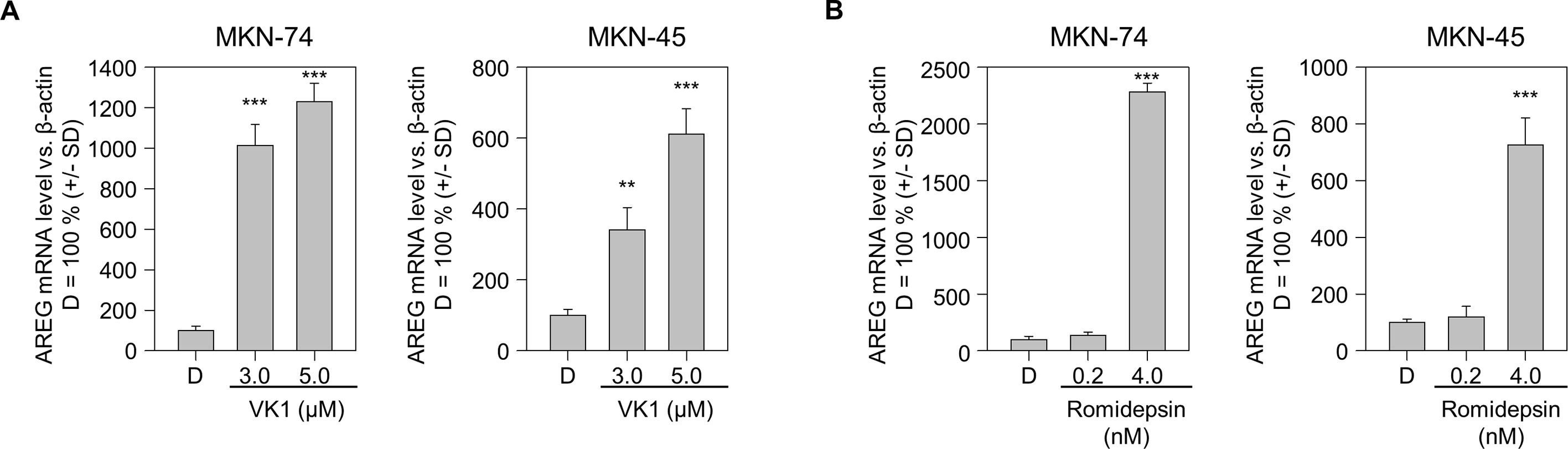

Supplement: Supplementary file 4 — Suppl. Fig. 4 Effect of different HDAC inhibitors on AREG mRNA level. The cell lines MKN-74 and MKN-45 were treated for 48 h with the class I HDAC inhibitors (A) VK1 and (B) romidepsin (Romidepsin) in two different concentration or DMSO as a vehicle control. Shown are the mean values of three independent experiments + S.E.M. [file mmc4.jpg]

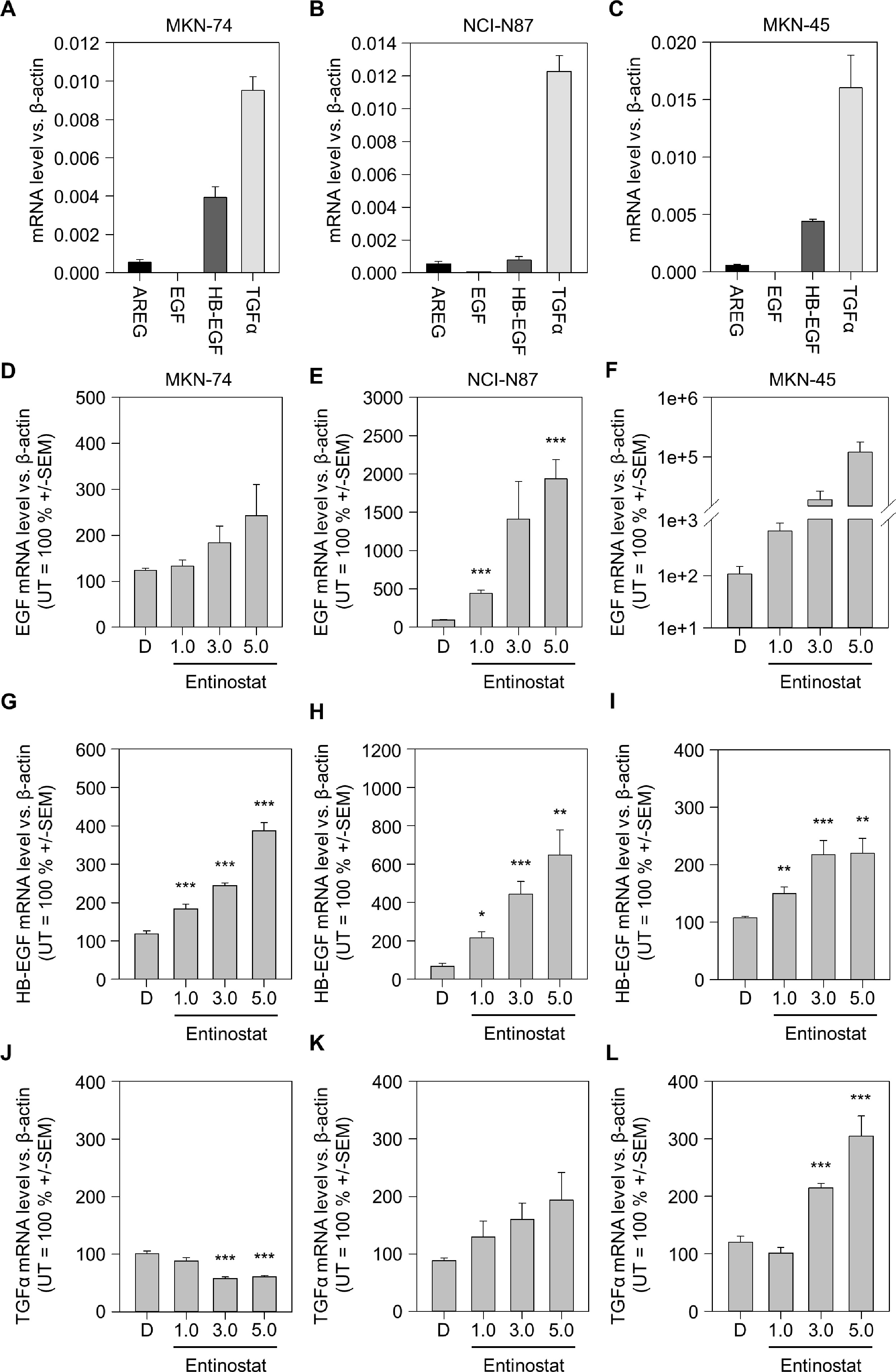

Supplement: Supplementary file 5 — Suppl. Fig. 5 Basal level and impact of entinostat treatment on various EGFR ligands in different gastric cancer cell lines. The basal expression of the EGFR ligands AREG, EGF, HB-EGF and TGF-α on mRNA level (upper A, B and C) was measured by qPCR. Additionally the changes in expression on mRNA level for the ligands EGF, HB-EGF and TGF-α after 48 h of entinostat treatment vs. vehicle control DMSO for the are shown. On display are the mean values of three independent experiments + S.E.M. [file mmc5.jpg]

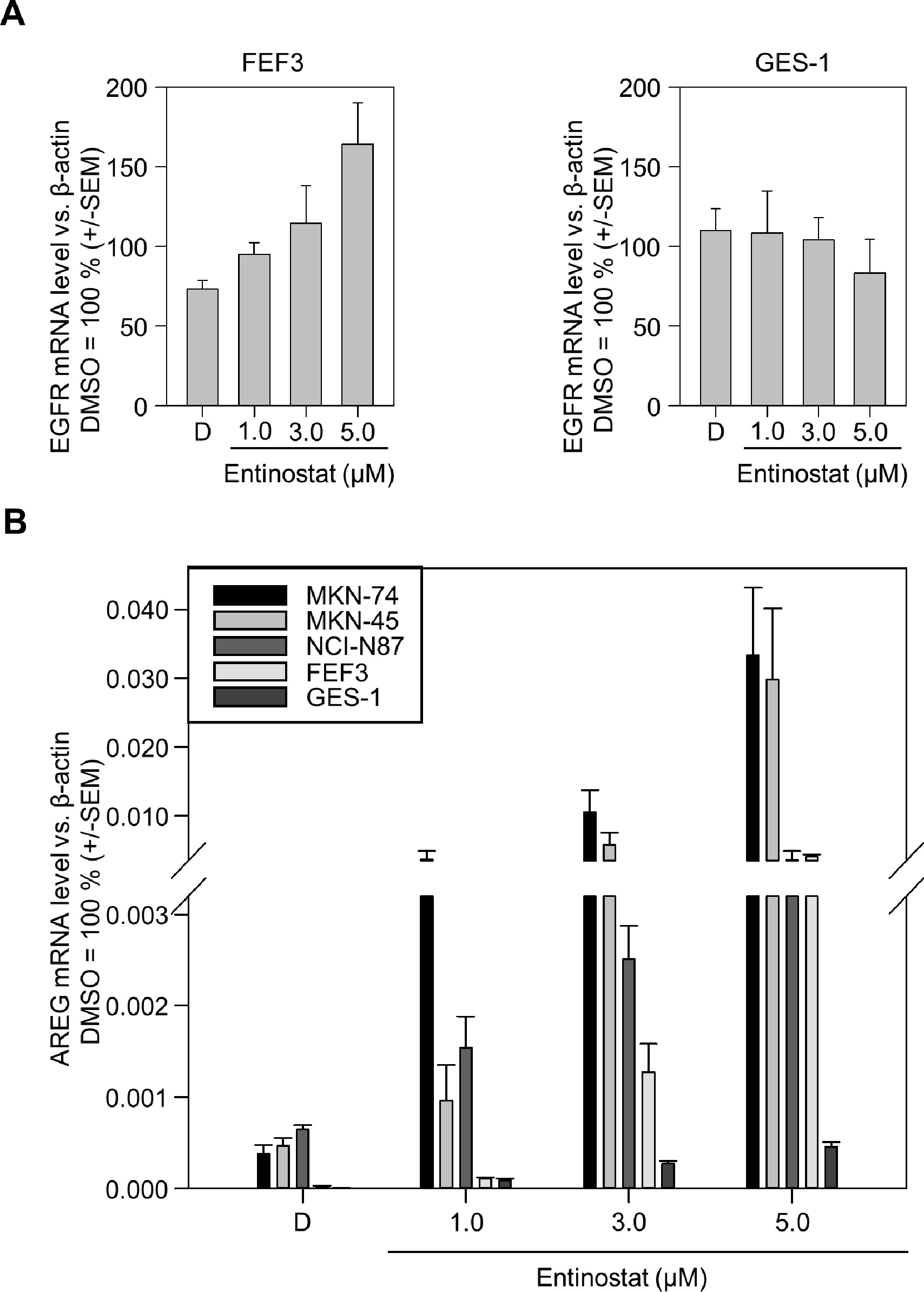

Supplement: Supplementary file 6 — Suppl. Fig. 6 Impact of an entinostat treatment on non-neoplastic cell lines. The fibroblast cell line FEF3 and the gastric epithelial cell line GES-1 were treated with entinostat or DMSO as vehicle control for 48 h. Changes of the mRNA-level of (A) EGFR were measured by qPCR compared to beta actin and normalized to an untreated sample. (B) AREG mRNA level of the non-malignant cell lines was normalized to beta actin only and compared to the cancer cell lines MKN-74, MKN45 and NCI-N87. All bars represent the mean of three independent experiments + S.E.M. [file mmc6.jpg]

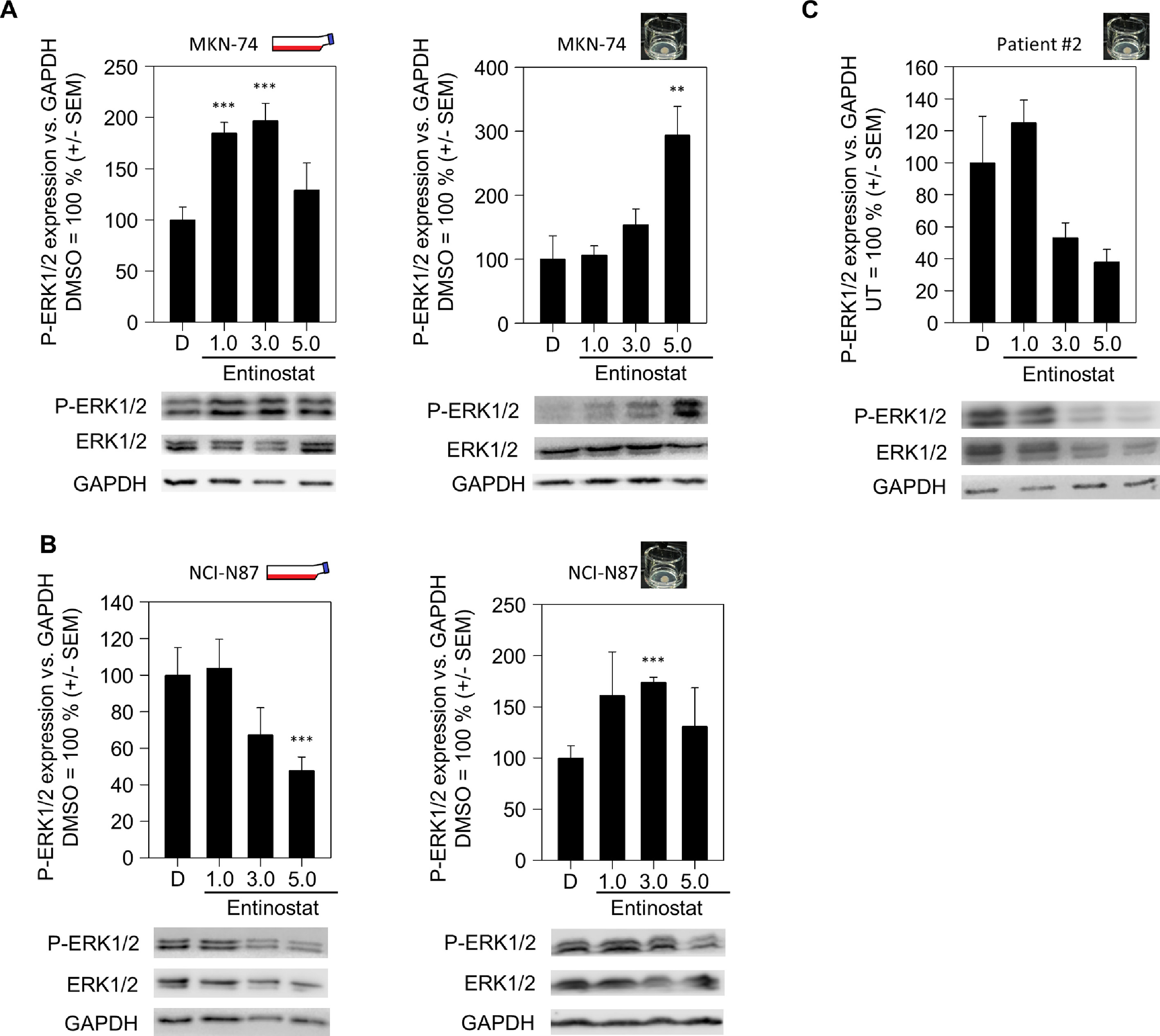

Supplement: Supplementary file 7 — Suppl. Fig. 7 Effect of entinostat on EGFR signalling via ERK1/2 phosphorylation. The cell lines (A, left) MKN-74 and (B, left) NCI-N87 were treated for 72 h with entinostat vs. vehicle control (DMSO), prior to analysis of protein samples by immunoblot. The bars represent the quantitation of phosphorylated ERK1/2 bands normalized to the loading control (GAPDH) from three experiments, with DMSO treated cells set to 100 %. Additionally, a representative Western blot is shown in each case. Results from 2D cell culture (A, B, left), tissue slice culture (A, B, right) and patient #2 PDX tissue slices (C) are shown, with bars giving the mean values of three independent experiments + S.E.M. [file mmc7.jpg]

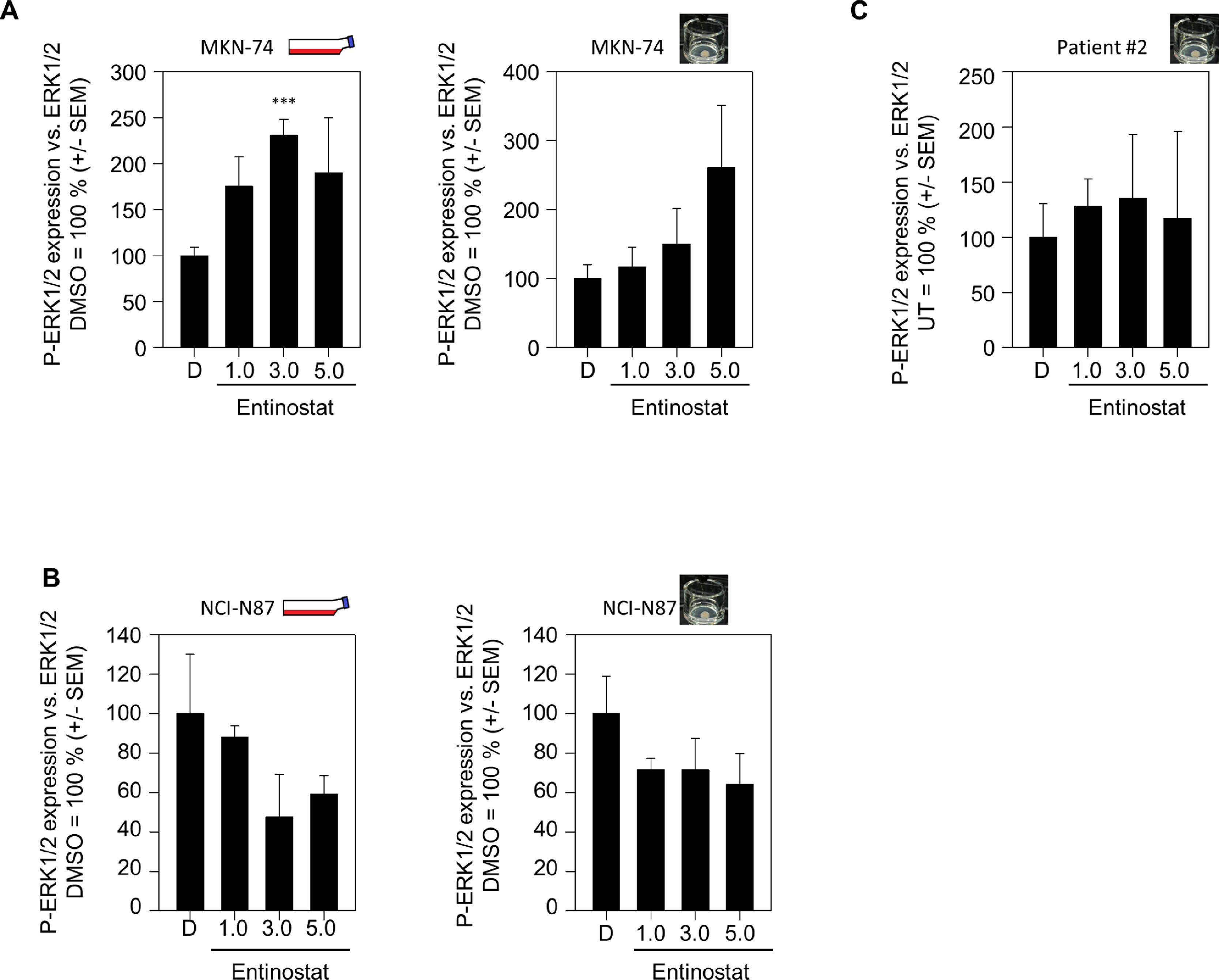

Supplement: Supplementary file 8 — Suppl. Fig. 8 Quantification of phosphorylated ERK in relation to total ERK after entinostat treatment. The cell lines (A, left) MKN-74 and (B, left) NCI-N87 were treated for 72 h and the tissue slices of (A, right) MKN-74, (B, right) NCI-N87 and (C) PDX material (patient #2) were treated for 72 h with entinostat or the vehicle control DMSO. Changes in expression were detected by Western blot. The mean values of 2-3 independent experiments + S.E.M. are shown. [file mmc8.jpg]
