# Supplementary material for: Acquired vulnerability against EGF receptor inhibition in gastric cancer promoted by class I histone deacetylase inhibitor entinostat
Source: Neoplasia. 2025 Jan 25;60:101121. doi: 10.1016/j.neo.2024.101121 (PMC11802376; doi:10.1016/j.neo.2024.101121)
Supplement: Supplementary file 9 [file mmc9.docx]

| **Gene** | **Primer** | **Nucleotide sequence (5’ – 3’)** |
| --- | --- | --- |
|  |  |  |
| Beta-actin | Forward | CCA ACC GCG AGA AGA TGA |
|  | Reverse | CCA GAG GCG TAC AGG GAT AG |
| AREG | Forward | TCC ATT CTC TTG TCG AAG TTT CT |
|  | Reverse | TGA TCC TCA CAG CTG TTG CT |
| TGF-Alpha | Forward | TGG CTG TCC TTA TCA TCA CAT GT |
|  | Reverse | CGG CAC CAC TCA CAG TGT TT |
| HB-EGF | Forward | TGG GGC TTC TCA TGT TTA GG |
|  | Reverse | CAT GCC CAA CTT CAC TTT CTC |
| EGF | Forward | AGT GCA TCC ACT TGC ACA AC |
|  | Reverse | CAA TTT GCA AAG TTT CTG CTC |
| EGFR | Forward | ACA CAG AAT CTA TAC CCA CCA GAG T |
|  | Reverse | ATC AAC TCC CAA ACG GTC AC |

Supplementary table 1: Primer sequences

Supplementary table 2: Immunoblot, FACS and immunohistochemistry antibodies used in this study, with dilutions and other specifications

| **Target protein** | **Clone** | **Dilution** | **Buffer (IHC)** | **Vendor, catalogue number** |
| --- | --- | --- | --- | --- |
| GAPDH | 1E6D9 | 1:5000 | - | Proteintech, 60004-1-Ig |
| AREG | - | 1:1000 | EDTA | Proteintech, 16036-1-AP |
| EGFR | H11 | 1:1000 | Tris-EDTA | ThermoFisher, MA5-13070 |
| P-ERK1/2 (T202/Y204) | D13.14.4E | 1:1000 | - | CellSignaling, 4370S |
| ERK1/2 | - | 1:1000 | - | CellSignaling, 9102S |
| Multi-rAb CoraLite® Plus 488-Goat Anti-Mouse Recombinant Secondary Antibody (H+L) | - | 1:500 | - | Proteintech, RGAM002 |
| Anti-mouse IgG (H+L), F(ab')2 Fragment (Alexa Fluor® 647 Conjugate) |  | 1:1000 |  | CellSignaling, 4410S |

Supplementary table 3: Patient / tumour characteristics for samples used for patient-derived xenograft models.

| **Patient number** | #1 | #2 |
| --- | --- | --- |
| **Gender** | Female | Male |
| **Age** | 81 | 77 |
| **Tumor type / grade** | Intestinal type, G2 | Intestinal type, G3 |
| **Staging** | pT3, pN1 (1/30), M0,  L1, V0, Pn1 | ypT3, ypN3 (16/29), pM1 (peritoneal), L1, V0, Pn0 |
| **Neoadjuvant**  **chemotherapy** | No | 4 cycles FLOT |
| **Additional information** | MSI-High |  |
